# Comparison of the diagnostic value of CBCT and Digital Panoramic Radiography with surgical findings to determine the proximity of an impacted third mandibular molar to the inferior alveolar nerve canal

**Published:** 2015

**Authors:** R Saraydar-Baser, M Dehghani-Tafti, A Navab-Azam, F Ezoddini-Ardakani, S Nayer, Y Safi, N Shamloo

**Affiliations:** * Department of Oral and Maxillofacial Radiology, Faculty of Dentistry, Ardabil University of Medical Sciences, Ardabil, Iran; ** Department of Oral and Maxillofacial Radiology, Faculty of Dentistry, Shahid Sadoughi University of Medical Sciences, Yazd, Iran; *** Department of Oral and Maxillofacial Radiology, Faculty of Dentistry, Alborz University of Medical Sciences, Karaj, Iran

**Keywords:** digital radiography, panoramic radiography, CBCT, mandibular canal, mandibular molar

## Abstract

**Background:** This study evaluated and determined the proximity of an impacted third mandibular molar (TMM) to the inferior alveolar canal (IAC) by using CBCT and digital panoramic radiography.

**Materials and Methods:** This descriptive-analytic research applied CBCT and panoramic radiographs for 60 subjects (28 men, 32 women). Subjects selected showed a close proximity about the TMM to the inferior nerve canal on panoramic radiographs; these subjects then received CBCT radiographs. The CBCT findings for the proximity of the TMM to inferior nerve canal used the outcomes of surgical findings as the standard of comparison.

**Results:** Eight cases showed positive surgical findings indicating vicinity of the third molar and the mandibular nerve canal. Only 13.3% of the cases in which panoramic views showed the proximity of the TMM and the IAC were confirmed during surgery. The result for CBCT radiographic diagnosis was 95%.

**Conclusion:** It can be concluded that CBCT is preferred over panoramic radiography to determine the proximity of the impacted TMM to the IAC. Narrowing of the mandibular canal or root canal, disconnection of root borders in panoramic radiography, and the inferior-lingual proximity of the tooth to the root in CBCT strongly indicated the close nearness of the impacted TMM to the IAC.

## Introduction

The extraction of an impacted TMM is a common minor operation in the maxillofacial region. Like other surgeries, this type can have the side effect of malfunction of the inferior alveolar nerve (IAN). It is necessary to precisely predict the vicinity of the third molar to the eIAN [**1**,**2**]. Although panoramic imaging offers comprehensive coverage and easy access, identifying the exact proximity of the impacted TMM to the IAC in patients is not possible; hence, it is essential to augment diagnosis using cone-beam computed tomography (CBCT) [**3**,**4**].

One side effect of impacted third molar tooth surgery is the malfunction of the IAN [**5**]. Such damage may cause paresthesia, hypoesthesia, and anesthesia of the lower lip. Its prevalence has been reported to be 4% to 8%; in less than 1% of cases, patients experience permanent numbness in that area [**6**-**9**]. This occurs because of the surgery in the area around the impacted molar root and the IAC results in exposure of or damage to the canal [**10**].

**Fig. 1:**
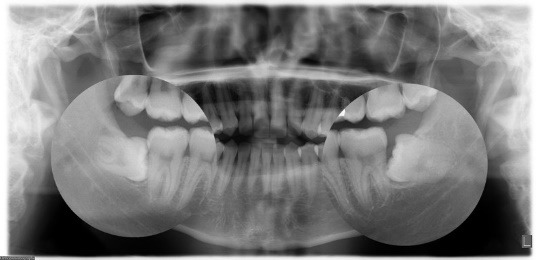
Panoramic radiograph

The proximity of the impacted third molar to IAN raises the risk of numbness up to 30% and may generate mental and social troubles for the patients [**11**,**12**]. This is also the reason of one of the very frequent complaints against maxillofacial surgeons in the coroner’s court and increases belief by the public that surgical negligence has occurred during surgery [**3**]. An extensive survey of the proximity of the impacted third mandible molar to the IAN is necessary before surgery. Panoramic radiography is the most common equipment used for pre-surgery evaluation of impacted third molars (**Fig. 1**).

Although this technique has gained prominence in third molar surgeries because it involves a low dose of radiation, comprehensive coverage, and simplicity of analysis and access, it has drawbacks. These include low sensitivity, 2D views, inability to distinguish bone thickness, distortion of dimensions and magnification of both the vertical and horizontal dimensions and production of ghost images on the reverse side. Sensitivity values of 24% to 64% and specificity values of 74% to 98% have been recorded for panoramic radiography [**3**-**5**]. This technique is gradually being replaced by CBCT, which allows 3D views of the anatomy with the least distortion at different angles [**11**]. The advantages of CBCT over CT include a decrease in the radiation surface, high-quality images, low scanning period, reduction in the radiation dosage to patient, and the decrease in metal artifacts in images [**2**].

Studies show that nerve damage is the most frequent side effect of surgery for the extraction of the third molar (4.4% to 8.1%). Paresthesias recorded in 1.3% to 5.3% of the cases because of the vicinity of the impacted tooth to nerves [**3**]. 

Atsuko et al. surveyed the positions of the lower jaw molars and the mandible canal by using CBCT. They concluded that data on the distance between the canal and the tooth provided by CBCT are effective for the evaluation of possible damage to the IAN. The great resolution and less radiation dosage allow the use of these images for TMMs. CBCT images in specific and standard conditions and the rating of a sufficient number of samples are listed as the advantages in the study [**12**]. 

Chu et al. studied the location of the mandibular canal relative to an impacted third molar of the lower jaw by using CBCT. Their results were based on panoramic evaluations and indicated the increased prevalence of proximity of the mandibular canal to roots of third molars in cases showing deep latency, narrow mandible canals, and samples showing white line radiopacity in the canal. They stated that the use of CBCT made it possible to carefully specify the location of the mandible canal and the root of the tooth. 

The current study compared the accuracy of panoramic radiography and CBCT with the surgical findings specifying the location of the impacted TMMs to the IAN. 

## Materials and Methods

This descriptive-experimental research was carried out by using a cross-sectional method. The subjects were chosen from patients awaiting surgery for removal of their third molars in the Department of Maxillofacial Surgery of the Dental department of Shahid Sadoughi University of Medical Sciences in Yazd, Iran. It is common to prescribe panoramic radiography for patients requiring impacted TMM surgery. All subjects chosen were patients at the same radiology center to provide a homogeneous sample. The panoramic radiographies were supplied by PlanmecaProMax (Helsinki, Finland) and were carried out under similar conditions (80 Kvp,12 mA,18 s). The 60 patients selected received panoramic radiographies that showed the existence of one or more signs of the vicinity of the root of the impacted tooth to the IAC. These signs were categorized in terms of their sensitivity as:

1. Darkening of the tooth root

2. Contraction of the tooth root 

3. Suspension of the white cortical line of the IAC 

4. Deviation or bending of the IAC 

5. Dark and bifid root apex

6. Island-shaped apex

7. Bending of the root 

8. Contraction of the IAC 

Patients who showed a gap between the tooth root and canal, for whom the root of the impacted tooth was not fully formed or who had lesions at the end of the apex were excluded from the study. Patients who had one or more radiographic signs were chosen for the study and were sent to obtain CBCTs. 

**Fig. 2:**
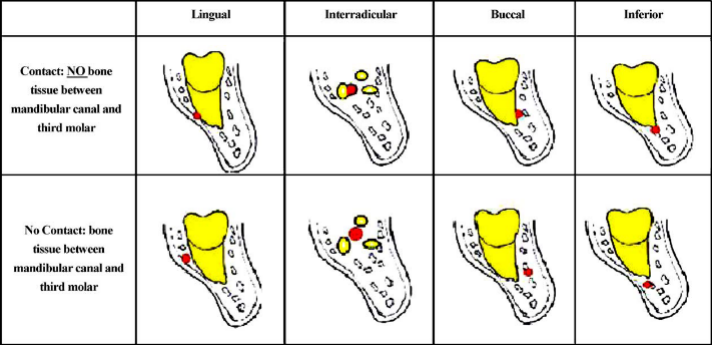
Schematic of CBCT evaluation of proximity of impacted TMMs to IAC

Before beginning, the reason behind the research and the advantages and disadvantages of the method were described to the subjects and written informed consent forms were collected from each. All subjects were scanned to observe and survey the condition of the tooth and IAC in 3D format. The 3D scans were taken by using CBCT (PromaxPlanmeca; Finland, Helsinki) under same conditions for exposure and resolution (80Kvp, 12 mA, 17 s). An observer surveyed the 3D radiographies by using 1 mm cuts of the image for axial, cross-sectional, and panoramic views. The criteria used to assess the CBCT radiographies are as it follows (**Fig. 2**):

• Lingual position of root to canal

• Buccal position of root to canal

• Inter-radicular position of root to canal

• secondary position of root to canal 

**Fig. 3:**
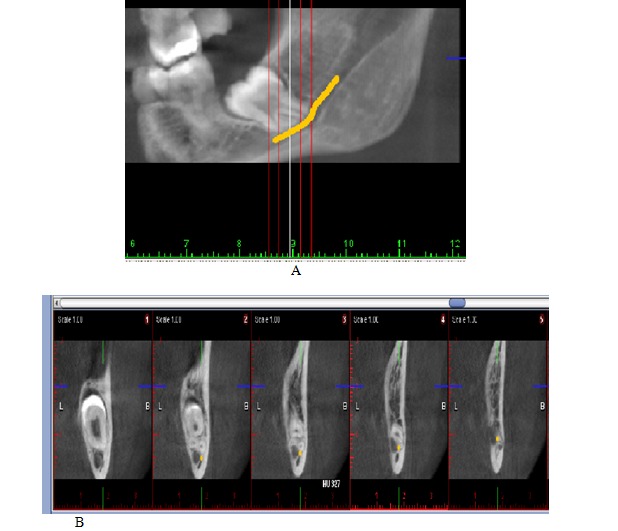
CBCT of left third molar: (A) 2D image; (B) multiplanar image.

CBCT images were taken by a maxillofacial radiologist (**Fig. 3**). Three checklists were prepared for each patient addressing the results of panoramic radiography, CBCT, and surgery. After scrutinizing the panoramic radiography and CBCT separately and at different times, the radiologist recorded his findings about the proximity of the tooth and nerve canal in the checklists (7 cases for panoramic radiography and 4 cases for CBCT). The radiologist was unable to consult with or compare his responses for the panoramic checklist while he was reviewing the CBCT images. The subjects were examined during surgery for signs of nerve involvement, bleeding, nerve exposure, and postoperative paresthesia. 

The surgeon completed his checklists by using the radiological results and from personal observation during surgery. The surgeon made the following observations during surgery:

1. Close proximity of root and nerve were observed as evidenced by a curve in the root or a nerve bundle near the root

2. No proximity of root to canal

3. Uncertainty about exposed area, which was obscured by bleeding

The present research compared the positive predictive value of panoramic radiography and CBCT and the diagnostic value of CBCT in specifying the proximity of the impacted TMM to the IAN. The results of the operation were then contrasted with the prior radiographic results. The data was compiled in SPSS 17 and analyzed by using the chi-square, Fisher’s exact, and Kappa tests. 

## Results

A whole of 28 men and 32 women took the role in this study. **Table 1** presents the results of the Fisher's exact examination for the relation of PPV by gender.

**Table 1:** Frequency allocation of surgical findings by gender

Surgical Findings / Gender	+		-		Total	
	Number	Percentage	Number	Percentage	Number	Percentage
Male	5	17.9	23	82.1	28	100
Female	3	9.4	29	90.6	32	100
Total	8	13.3	52	86.7	60	100
P-value= 0.454 Fisher’s Exact Test						

The PPV for panoramic radiography was 13.3% compared to the surgical results. This showed that surgery confirmed only 13.3% of the possible proximity of the impacted third molar tooth to the IAC of the lower jaw as assessed while using panoramic radiography. The results determined that the diagnostic value of CBCT correlated much more highly with the results of surgery for diagnosing possible vicinity of the impacted third molar tooth to the IAC of the lower jaw of subjects who had positive panoramic results (**Table 2**). 

**Table 2:** Frequency distribution of surgical findings by side involved

Surgical Findings / The side involved	+		-		Total	
	Number	Percentage	Number	Percentage	Number	Percentage
Right side	6	14.6	35	85.4	41	100
Left side	2	10.5	17	89.5	19	100
Total	8	13.3	52	86.7	60	100

The accuracy of CBCT was 100%, which shows the excellent ability of this technique for diagnosing positive cases (false + real positive). The ability of CBCT of specifying and diagnosing negative cases was 94% (false positive + real negative). The PPT for this technique was 72%, indicating a significant positive predictive value. It implies that 72% of 100 cases diagnosed as positive by this technique were real positive by using the results of surgical findings as the standard of comparison. This factor is significantly better than for panoramic radiography. The PPV was 3.3% for panoramic radiography, which is outstandingly low and unreliable (real positive/ real positive + false positive). The negative predictive value of CBCT was 100%, showing that the NPV of this technique is reliable. All cases were congruous with the results of surgical findings as the standard of comparison (real negative/ real negative + false negative).

The precision of the study was 95%; in 95% of cases, the surgical results were congruous with CBCT results. The characteristic value of CBCT radiography was evaluated by using this index. The agreement of CBCT results with the surgical results was evaluated at a Kappa of 0.813, which is important at p = 0.001. This recommends that the results of CBCT tests were in conformance with the operational outcomes for those patients having positive panoramic radiographies (**Table 3**).

**Table 3:** Diagnostic value of radiographic findings of CBCT

Surgical Findings / CBCT findings	+		-		Total	
	Number	Percentage	Number	Percentage	Number	Percentage
+	8	13.3	3	5	11	18.3
-	0.00	0.00	49	81.7	49	81.7
Total	8	13.3	52	86.7	60	100
P-value= 0.000 Measure of Agreement Kappa						

Seven factors used in previous studies were employed to evaluate the panoramic radiography to determine the proximity of an impacted third molar to the IAC. Only 3 out of 7 factors showed a meaningful accordance with the outcomes of operation. These factors were deflection and curvature of the root, dark bifid root apex near the nerve, and an island-shaped apex; they showed a significant agreement with the results of surgical findings as the standard of comparison atp = 0.022, p = 0.027 and p = 0.007, respectively.

When these 3 factors were surveyed by using panoramic radiography, the detection of the proximity of impacted third molars to the IAC of the below jaw increased significantly. The examination showed that the correspondence between the factors and the surgical results was not meaningful. The frequency distributions of the 3 factors are shown in **Table 4**. The first factor, dark bifid root apex, had the largest frequency. The lack of cortical borders of the alveolar canal, narrow nerve canal, and root apex deflection was not seen in panoramic radiography (**Table 4**).

**Table 4:** Frequency distribution of determining factors in panoramic radiography

Surgical Findings / Panoramic findings	+		-		Total		P-value
	Number	Percentage	Number	Percentage	Number	Percentage	
Interruption of white line of the mand. canal wall	5	62.5	30	57.7	35	58.3	0.000
Darkening of the root	1	12.5	18	34.6	19	31.7	0.416
Diversion of the mand. canal	0	0	0	0	0	0	-
Narrowing of the mand. canal	7	87.5	18	34.6	25	41.7	0.007
Narrowing of the roots	0	0	22	42.3	22	36.7	0.022
Deflection of the roots	3	37.5	3	5.8	6	10	0.027
Fisher’s Exact Test							

Four factors were used to evaluate CBCT: lingual, buccal, intra-radicular, and inferior positions of the root relative to the canal. The proximity of the impacted TMM to the IAC increased only when observing the inferior and lingual factors simultaneously and was statistically meaningful at p = 0.000 (**Table 5**).

**Table 5:** Frequency distribution of determining factors in CBCT radiography

Surgical Findings / CBCT	+		-		Total		P-value
	Number	Percentage	Number	Percentage	Number	Percentage	
1	7	87.5	26	50	33	55	0.063
2	0	0	10	19.2	10	16.7	0.330
3	4	50	9	17.3	13	21.7	0.059
4	4	50	43	82.7	47	78.3	0.059
5	3	37.5	17	32.7	20	33.3	0.000
6	0	0	10	19.2	10	16.7	0.330
Fisher’s Exact Test							

## Discussion

It is necessary for a surgeon to use radiography to determine possible difficulties arising during surgery and prepare for them before beginning surgery for an impacted third molar. There exists the possibility of damage to the sinus of the upper jaw or alveolar canal of the lower jaw during impacted tooth surgery [**13**]. Although several studies have surveyed the accuracy of panoramic and tomographic radiography, variables related to the risk of harm to the alveolar nerve have not been comprehensively studied [**14**]. 

The present research evaluated the diagnosis of close vicinity of the mandibular canal to the third impacted mandible molar by panoramic radiography by using CBCT images. Variables related to heightened risk of two-structure relatedness were identified [**15**]. Disorders of the IAN result from damage to sensory tissue; if the tooth and mandible canal are in close proximity, the risk increases. This lesion may be temporary, but could become permanent if scar tissue develops after surgery. The size of the patient dose in CBCT is lower than for a CT scan [**16**]. Studies have shown that CBCT is a suitable device to diagnose the closeness of the mandibular canal to prevent damage to it and its neurovascular bundle. Its diverse advantages recommend it for application in tooth surgery. 

Pawelzik et al. compared panoramic radiography and volumetric CT to study the impacted third mandible molars before surgery. They scanned 10 patients with impacted TMMs by using panoramic radiograph and found a close proximity of the tooth to the IAN. Five oral surgeons analyzed a number of anatomic factors. In 90% of the cases, volumetric CT (VCT) images facilitated the diagnosis of the proximity of the impacted third molar to other anatomical features. In 70% of the cases, the vicinity of the tooth apex to the nerve could be diagnosed by using VCT [**17**]. It has been stated that panoramic radiography and VCT are not adequate for diagnosis on their own and should be used together. The authors reported that, if an experienced radiologist is available to explain the panoramic radiography, VCT is not necessary [**18**-**20**]. 

The current study showed that panoramic radiography failed to correctly diagnose the relation between these two structures on its own. The influence of multiple observers for radiographic accuracy was eliminated by using only one observer and the reliability of the study increased. Several investigations have found that the factors used in panoramic radiography are better related to the proximity of the alveolar nerve to the impacted third molar of the lower jaw. Albert et al. compared panoramic radiography with conventional tomography to study the vicinity of the impacted third molar and the mandibular canal. They analyzed risk factors in the perception of close proximity of the tooth to the nerve and determined the topography of nerve to the mesial and distal roots. Their results determined that the darkening of the root was the most common factor isolated in panoramic radiography; in 13 out of 14 patients showing this sign, the third molar was in close vicinity to the nerve. In 4 out of 5 patients showing an island-shaped apex, the third molar was in close vicinity to the nerve. A dark bifid root apex and deflection of the root apex did not indicate a close proximity of the tooth root to the mandibular nerve. The performance of tomography versus panoramic radiography was not discussed [**21**]. 

Tantanapornkul et al. compared panoramic radiography and CBCT to evaluate the topographic proximity of an impacted third molar tooth to the mandibular canal. They considered 4 factors for the proximity of the nerve to the tooth: lack of continuity of mandibular canal; root darkening; mandibular canal deflection; and reduction of the root. The existence or nonexistence of a direct relationship between root and nerve were the criteria for CBCT. After the analysis of the radiographs, patients underwent surgery and the results determined during surgery were recorded. After surgery, patients were examined for the existence of paresthesia. The results revealed that every factor for panoramic radiography was related to the exposure of the nerve; hence, these factors effectively predicted the risk of harm to the nerve. The lack of continuity of the mandibular canal was introduced as the most important diagnostic factor. Specificity was 93% and sensitivity was 77% for CBCT, 70%, and 63% for panoramic radiography, respectively. This showed that CBCT outperformed panoramic radiography [**22**]. 

The frequency of 7 factors and their significance or non-significance was calculated by comparison with the results of operation. Three factors were found to have a meaningful relationship with the results of operation. Results showed that 3 out of 7 evaluations of panoramic radiography factors (diversion or bent IAC, island-shaped apex, and dark bifid root apex) had a meaningful relationship with results of operation as the standard. These were significant at p = 0.022, p = 0.027, and p = 0.007, respectively.

When these factors were found in panoramic radiographs, the probability of the close vicinity of the impacted third molar tooth of the below jaw to the IAC increased significantly. There were no significant relationships found between radiographs of the disruption of the white cortical line of inferior alveolar, root deflection, and narrowing of the IAC. Studies have considered factors such as different numbers of observers, their specialties, the method of scoring of data, and results of surgery results in their research methods. The exposure during surgery and the surgeon assessment were considered the guidelines for evaluation. In other cases, paresthesia was considered for the vicinity of the two structures [**23**-**29**]. Valmaseda-Castellón et al. showed that IAN injury might ensue after lower third molar operational extraction [**24**].

Tantanapornkul et al. surveyed the results of CBCT and panoramic radiography to assess the closeness of the mandibular canal to an impacted third molar. Patients with impacted third molars of the lower jaw were scanned by panoramic radiography prior to surgery. The surgeons were asked to record all tooth extraction details and neurovascular exposure during tooth extraction. Patients for whom there was doubt about neurovascular exposure were dismissed from the research. Seven days after surgery, the side effects of third molar surgery of patients were recorded. Ten patients showed the side effects; patients with exposed neurovascular bundles showed notably higher side effects compared to other patients. The sensitivity of CBCT was 93%, which was notably greater than for those receiving only panoramic radiography. It was concluded that the CBCT was more effective in predicting neurovascular exposure after surgery for an impacted third molar than panoramic radiography. Moreover, its application under clinical conditions to evaluate impacted third molar pre-surgery had several advantages. Since identifying neurovascular exposure was done by the surgeon during surgery, the possibility exists that some areas were overlooked and these results showed the low specificity of images [**21**]. This was a restriction of the research. The present study employed observers, which had several advantages. 

Gaeminia et al. evaluated the proximity of impacted third molars to the mandibular canal by using CBCT and panoramic radiography. Their results revealed no significant relation between exposed IAN and nervous disorders after surgery by gender, place of surgery or third molar angle. They found no meaningful difference between these two techniques for the prediction of the risk of nerve exposure; however, the lingual location of the mandibular canal was notably correlated to IAN nerve damage. Three cases using panoramic radiography were significantly related to IAN nerve damage. CBCT sensitivity was 96% and specificity was 23% [**18**-**22**]; hence, they found that the diagnostic precision of panoramic radiography and CBCT were the same. The benefits of this study were the random viewing of panoramic radiographic images and CBCT, internal agreement of viewers for both techniques, and evaluation by one observer. The sensitivity and specificity of both CBCT and panoramic radiography have been reported differently in various studies; for example, a sensitivity of 96% and specificity of 27% have been announced in a similar study.

In the current study, the sensitivity of CBCT was 100%, which indicates its effectiveness in diagnosing positive cases. Its specificity for diagnosing and identifying negative cases was 94%, which was lower than its sensitivity.

## Conclusion

This study confirmed that CBCT is the most accurate method of radiography for the determination of the proximity of impacted third molars of the lower jaw and the IAC. The results indicated that 3 of 7 factors used to evaluate panoramic radiography (diversion or bending of the IAC, dark bifid root apex) notably matched with the operational results used as the standard. The CBCT diagnostic value was 95% in this study, indicating that, in 95% of cases, the results of operation were the same as the predictions from CBCT. The results of CBCT evaluation increased for simultaneous observation of the inferior-lingual relation to confirm the proximity of the impacted third molar of the lower jaw to the alveolar canal. 

**Conflict of interest**

The authors declare that they have no conflict of interest.
